# User Identification from Gait Analysis Using Multi-Modal Sensors in Smart Insole

**DOI:** 10.3390/s19173785

**Published:** 2019-08-31

**Authors:** Sang-Il Choi, Jucheol Moon, Hee-Chan Park, Sang Tae Choi

**Affiliations:** 1 Department of Computer Science and Engineering, Dankook University, Yongin 16890, Korea; 2 Department of Computer Engineering and Computer Science, California State University Long Beach, Long Beach, CA 90840, USA; 3 Department of Internal Medicine, Chung-Ang University, Seoul 06984, Korea

**Keywords:** multi-modal feature, gait analysis, multi-modal sensors, smart insole, user identification, wearable sensor, linear discriminant analysis

## Abstract

Recent studies indicate that individuals can be identified by their gait pattern. A number of sensors including vision, acceleration, and pressure have been used to capture humans’ gait patterns, and a number of methods have been developed to recognize individuals from their gait pattern data. This study proposes a novel method of identifying individuals using null-space linear discriminant analysis on humans’ gait pattern data. The gait pattern data consists of time series pressure and acceleration data measured from multi-modal sensors in a smart insole used while walking. We compare the identification accuracies from three sensing modalities, which are acceleration, pressure, and both in combination. Experimental results show that the proposed multi-modal features identify 14 participants with high accuracy over 95% from their gait pattern data of walking.

## 1. Introduction

Gait patterns contain much information about human physical activity. Problems in gait can be entail not only musculoskeletal disorders, such as joint deformation [1], but also mental disorders, such as intellectual disabilities [2], dementia [3], and depression [4]. Given its insightful outcomes, the analysis of gait patterns has received abundant attention in various fields including health care, sports performance analysis, and behavior analysis [5,6,7].

Gait pattern analysis comprises a sensor module for acquiring data and an application module for analyzing the data [8]. Different types of sensors are utilized in gait analysis, for instance video recorders [9], electromyography sensors [10], pressure sensors [11], accelerometers [12,13], and gyroscopes [14,15]. Initially, the gait pattern analyses were conducted in restricted environments because of the size of sensors, the inconvenience of installing sensors, and other limitations. However, these days, such restrictions are alleviated using embedded sensors in wearable devices such as smart watches, fitness trackers, and smart insoles [16].

Several methods for analyzing gait patterns using data from diverse sensors have been proposed. In [17], straight and curved walking patterns were distinguished using a pressure sensor and a gyroscope. In [18], gait data for walking, sideways walking, and running were collected using only an accelerometer, and in-plane displacement was estimated.

Gait pattern analysis using machine learning approach has also been investigated. In [19], inertial measurement units (IMUs) attached to thigh and knee were used to measure kinematic data. In [20,21], spatiotemporal gait features, such as stride length, cadence, stance time, and double support time, were estimated using pressure-sensitive GaitRite walkways or foot switches. Then, gait patterns of patients with Parkinson’s disease were analyzed using support vector machine, random forest [20], or a mixture model [21]. In [22], gait types and behaviors were classified by applying a decision tree and an artificial neural network [23] to data collected by attaching different kinds of sensors such as accelerometers, gyroscopes, and humidity sensors to eight body parts.

Statistical and probability-based methods have been proposed to analyze walking patterns as well. In [24], gait phase classification was performed by applying a hidden Markov model to IMU data acquired from the legs and switches attached to the sole. Likewise, in [25,26], hidden Markov models were used for identifying users and determining the walking style from IMU data, respectively. Overall, the analysis of gait patterns, including the above-mentioned methods, has mainly been carried out for classifying gait types or for diagnosing diseases such as Parkinson’s disease or strokes by identifying abnormal gait patterns.

As gait patterns exhibit specific characteristics according to the individual, they can also be used for user identification if used along with other biometric techniques, such as face or fingerprint recognition. Existing gait analyses for biometrics have mainly been conducted using video sequences [9]. However, such approaches require the user to be the only individual in front of the camera, and their accuracy may vary depending on the relative position of the camera. Therefore, these methods provide limited user identification in real-world measurement environments. Besides motion analysis, wearable sensors have been utilized for user identification. In [27], data were collected from five IMUs placed on the chest, lower back, right-hand wrist, knee, and ankle of users. Identification was achieved using a predictive model based on a convolutional neural network with time- and frequency-domain data. In [28], IMU data were gathered using the sensors embedded in smartphones, which were carried by users in their front trouser pockets. Users were recognized using a mixture model based on a convolutional neural network and a support vector machine. In [29], besides IMU data from sensors within the shoes, pressure and flexion data were collected from insole sensors, and users were identified by a cascade neural network. However, these methods use few types of sensors, place sensors at multiple body parts, or require a long period of time for gathering data.

**Figure 1:**
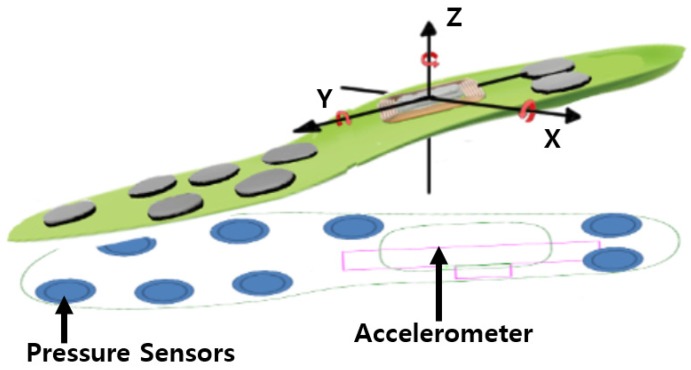
Sensor structure of the smart insole, “FootLogger”.

In this paper, we propose a method to identify users by using multi-modal sensor data acquired through a smart insole. For data collection, we used the pressure sensors and accelerometers of the FootLogger smart insole (Figure 1) [8]. The data acquired from each sensor in the insole during walking were transmitted to a smartphone via Bluetooth. While existing gait analyses using wearable sensors identify gait types, we attempted to perform user identification using gait data through discriminant analysis. Since the proposed method uses wearable sensors, it can be applicable to any type of user environment, for instance multiple users in a public place. In addition, the wearable sensor data (i.e., pressure and accelerometer) demand a low computational cost compared to video processing and thus can achieve real-time operation.

The proposed method consists of a preprocessing stage for extracting discriminant features and a classification stage for identifying users. During preprocessing, the measured data are converted into a form suitable for discriminant analysis to conduct user identification. Gait patterns can vary even for the same user depending on several factors, for example walking speed is typically dependent on the user’s mental and physical condition. The high variability of intrapersonal gait patterns may hinder feature extraction for user identification. Thus, during data preprocessing, we segmented the series of gait data into individual steps, then they were normalized in terms of their length to eliminate speed variability [30]. Thus, during data preprocessing, we segmented the series of gait data into individual steps, then they were normalized in terms of their length to eliminate speed variability [30]. In addition, random noises were added to the normalized data to prevent rank deficiency during feature extraction.

Since the proposed method is intended to be used with wearable devices, such as a smart insole, gait pattern features were extracted using a dimensionality reduction method with low computational resource requirements such that it was applicable to mobile systems. As insoles for both feet generate 16 pressure and six acceleration signals in real time, the obtained walking data were high-dimensional. Therefore, we extracted discriminant features for user identification by using the null-space linear discriminant analysis (NLDA) method [31], which effectively handles high-dimensional data, such as images. We applied NLDA to pressure and acceleration data to construct feature spaces and obtained single-modal feature vectors for each data type. Then, we evaluated the discriminative information of each feature based on the Laplacian score [32] and constructed multi-modal features for user identification by rearranging the features according to their discriminative information. Experimental results using measurements from 14 participants during walking demonstrated the high user identification performance of the proposed method.

The remainder of this paper is organized as follows. In Section 2, we detail the smart insole for walking data acquisition and the preprocessing stage. In Section 3, we describe the extraction of the single-modal features for each sensor data type and construction of the multi-modal feature vector for identification. In Section 4, we present the experimental results regarding user identification. Finally, we draw conclusions in Section 5.

## 2. Data Acquisition and Preprocessing

### 2.1. Gait Data Acquisition

We used the FootLogger smart insole for gait data collection (Figure 1). The insole is equipped with eight pressure sensors and a triaxial accelerometer [30]. Three pressure sensors are placed on the front left side, three others on the front right side, and the remaining two on the heel. Each pressure sensor retrieves values of 0, 1, and 2 depending on intensity, where 0 indicates no pressure, that is, the foot is off the ground, whereas values of 1 and 2 indicate increasing pressure at the location of foot contact with the ground. The sensors in both feet synchronously acquire data at a sampling rate of 100 Hz. These measured data are transmitted to a database server through a Bluetooth application using an Android smartphone.

### 2.2. Data Normalization and Regularization

Gait data are time series signals that reflect characteristic repetitive patterns. Hence, we extracted the features of gait patterns from the gait cycles, which corresponded to the minimum period of repetition. In general, a gait cycle [33] comprises the movement from the moment one foot touches the ground to the moment where it leaves the ground and returns to the ground. The gait cycle is usually divided into two stages, namely the stance phase, where the foot touches the ground, and the swing phase, where the foot leaves the ground. More detailed models consider seven stages, namely heel strike, foot flat, mid-stance, heel off, toe off, mid-swing, and late swing.

**Figure 2:**
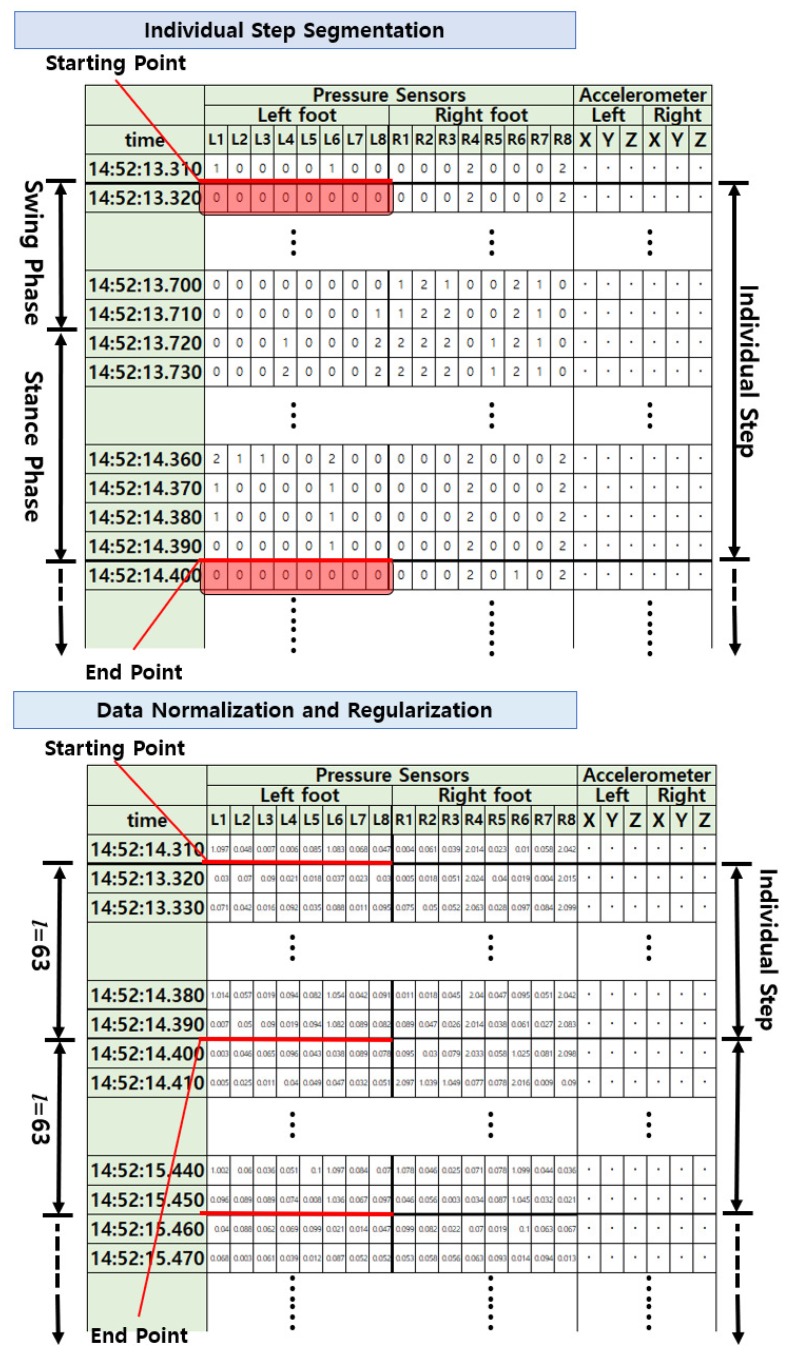
Preprocessing for gait pattern analysis.

We first detected the starting and ending points of the gait cycle according to the swing phase onset, in which all the pressure sensors on the insole of one foot retrieve a value of zero. Then, the continuously-measured gait data were divided into individual steps, each corresponding to one gait cycle. For each of the pressure sensors and accelerometers, the data points of individual steps were stored in matrix form by arranging the sensor values of both feet side by side along time axis *l*. Hence, each column represents a sensor, and the rows indicate time (Figure 2). As a result, pressure data from the eight sensors and triaxial acceleration data of both feet were stored in matrices with 16 and six columns, respectively.

Although the walking speed may be a distinguishing the characteristics of each person, it can also be a factor that increases within-class data variability, because one person can walk at a varying pace according to different conditions. Therefore, we normalized the gait data to a fixed period l=63 per individual step to eliminate the variability of gait cycle length [30]. Hence, the normalized values of the pressure and acceleration sensor arrays per step were given by matrices of 63×16 and 63×6, respectively.

Most statistical-based feature extraction methods have specific scattering matrices resembling the data covariance matrix to define their objective functions. Therefore, to utilize these methods, we converted the sensor data from matrices into vectors of pressure (1008×1) and acceleration (378×1) per step using lexicographic ordering. On the other hand, as every step was divided according to the swing phase, some elements of the vector became zero in all the samples, which may lead to rank deficiency during calculation of the covariance matrix. To prevent this instability problem related to eigenvalue decomposition, we performed regularization [34] by adding random numbers between zero and 0.1 to the data values. Figure 2 shows the original and preprocessed data for gait pattern analysis.

## 3. Multi-Modal Features for Identification

### 3.1. Discriminant Feature Extraction

As the FootLogger sensors measure data every 0.01 s, the resulting gait data were a high-dimensional vector. Therefore, we extracted features of gait data using NLDA, which avoids the small sample size problem [35] that occurs when dealing with high-dimensional data in supervised machine learning for classification. NLDA is a variant of the linear discriminant analysis (LDA) [35] and proceeds as follows. By projecting samples into the null space of the within-class scatter matrix, NLDA aggregates samples from the same class into one place and distributes the distance between the means of samples in different classes to create the feature space. NLDA effectively handles high-dimensional data due to securing the null space of the within-class scatter matrix.

Pressure and acceleration gait data exhibit different properties in terms of content and format. Besides the different physical factors being measured, the pressure sensor retrieves three discrete quantification levels, whereas the acceleration data have a continuous property (in spite of being sampled). Thus, we separately applied NLDA to the pressure and acceleration data to extract single-modal features and then evaluated the discriminative power of each feature to construct a multi-modal feature vector for identification.

Let *C* and *n* be the number of users to be classified and the dimension of the preprocessed data samples, respectively. The sensor data can be represented as $x^{S}\in R^{n}$, with *S* being *P* for pressure and *A* for acceleration. If the number of samples belonging to each class is $N_{i}$, $S_{W}^{S}=\sum_{i=1}^{C}\sum_{x_{j}^{S}\in c_{i}}\left(x_{j}^{S}-\mu_{i}^{S}\right){\left(x_{j}^{S}-\mu_{i}^{S}\right)}^{T}$, where $x_{j}^{S}$ is the $j^{\mathrm{th}}$ sample belonging to class $c_{i}$ and $\mu_{i}^{S}$ is the sample mean of $x_{j}^{S}$. In addition, inter-class scatter matrix $S_{B}^{S}=\sum_{i=1}^{C}N_{i}\left(\mu_{i}^{S}-\mu^{S}\right){\left(\mu_{i}^{S}-\mu^{S}\right)}^{T}$, where $\mu^{S}$ is the mean of the total samples. In discriminant analysis using $S_{W}^{S}$ and $S_{B}^{S}$, the null space of $S_{W}^{S}$ has a very high discriminative power because it gathers the samples belonging to the same class into one point. To maximize discrimination between classes, NLDA projects the samples in the null space and finds the feature space where the variance between the means across classes is maximized through the following objective function:

$$W_{Opt}^{S}=argmax_{\left|W^{T}S_{W}^{S}W\right|=0}\left|W^{T}S_{B}^{S}W\right|,$$

where $W_{Opt}^{S}$ is a projection matrix composed of $n^{\prime }$ projection vectors $w_{n^{\prime }}^{S}$, and feature vector $y^{S}$ for sample $x^{S}$ can be obtained as:

(1) $$y^{S}\left(\in R^{n^{\prime }\times 1}\right)={\left(W_{Opt}^{S}\right)}^{T}x^{S},y^{S}\left(=\left[y_{1}^{S},y_{2}^{S},\ldots ,y_{n^{\prime }}^{S}\right]\right)={\left(W_{Opt}^{S}\right)}^{T}x^{S}.$$

### 3.2. Multi-Modal Feature Vector Construction

Feature vector $y^{S}$ extracted from each sensor is composed of C−1 features, but not all of them evenly contribute to classification. The discriminative power of each feature is reflected by the corresponding eigenvalue of the projection vector, and projection matrix $W_{Opt}^{S}$ generally has a projection vector with a large eigenvalue. However, feature evaluation based on eigenvalue comparison is valid only in the same sensing mode. Therefore, we determined the discriminant power of all the features extracted from each sensor’s data by using feature selection and constructed a multi-modal feature vector with the most representative features from each sensing mode.

There are various ways to evaluate feature contribution, from which we selected the Laplacian score [32], as it measures the discernibility of features in a supervised way by determining discriminability based on local geometric structures. We first merged all features $y_{t}^{P}$ and $y_{t}^{A}$ (*t* = 1, …, C-1) for each sensor into a candidate vector $y^{candi}=\left\{y_{1}^{P},\ldots y_{C-1}^{P},y_{1}^{A},\ldots ,y_{C-1}^{A}\right\}$ for multi-modal feature vector and calculated the Laplacian score of each feature. To to this, we defined nearest-neighbor graph *G* with the number of training data (*N*) and weight matrix $M^{W}$ [32]. If two candidate vectors $y_{i}^{candi}$ and $y_{j}^{candi}$ corresponding to the $i^{\mathrm{th}}$ and $j^{\mathrm{th}}$ nodes, respectively, belong to the same class, an edge is placed between them. For linked nodes, $M_{ij}^{W}$ is $e^{-\frac{\left|\right|y_{i}^{candi}-y_{j}^{csndi}{\left|\right|}^{2}}{m}}$ (where *m* is a user parameter set to two), and $M_{ij}^{W}$ is zero otherwise. By letting $f_{r}$, *D*, and 1 be $\left[f_{r1},f_{r2},\ldots ,f_{rN}\right]$, $diag(M^{W}1)$, and ${\left[1,\ldots ,1\right]}^{T}$, respectively, the Laplacian score $LS_{r}$ for the $r^{\mathrm{th}}$ feature is calculated as [32]

(2) $$LS_{r}=\frac{{\tilde{f}}_{r}^{T}L{\tilde{f}}_{r}}{{\tilde{f}}_{r}^{T}D{\tilde{f}}_{r}},$$

where ${\tilde{f}}_{r}=f_{r}-\frac{f_{r}^{T}D1}{1D1}1$ and $L=D-M^{W}$. Features retrieving larger Laplacian scores were selected to construct multi-modal feature vector $y^{Mul}$, which was used as the input to the classifier for user identification.

**Figure 3:**
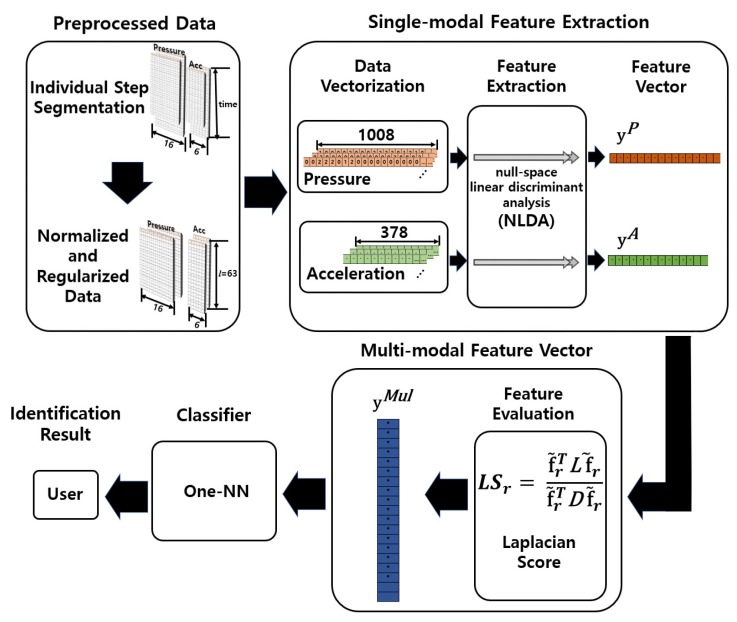
Procedure of the proposed method for user identification.

The complete procedure of the proposed method is summarized as follows (Figure 3):

1. Data measured from pressure sensors and accelerometers corresponding to continuous walking were divided into individual steps based on the swing phase determined from pressure data.
2. Data normalization was performed for every individual step to have the same time length, and regularization was performed for discriminant analysis.
3. For each type of sensor, single-modal features were extracted using NLDA from the preprocessed data.
4. The Laplacian score of each feature was calculated to evaluate its discriminative power, and a multi-modal feature vector was constructed by sequentially selecting highly-discriminant features.
5. The resultant multi-modal features were employed for user identification.

## 4. Experimental Results

To evaluate the performance of the proposed method, we measured gait data using the FootLogger insole from 14 adults aged between 20 and 30 years. The data were measured while participants walked for three minutes. The gait data from the 14 subjects retrieved 2295 individual steps by following the preprocessing presented in Section 2. From the samples, 700 steps were randomly selected, and three samples for each subject were used for training (total of 42 samples), while the remaining 658 samples were used for testing. As a result, we obtained a total of 2295 samples of individual steps for 14 subjects. To determine the number of steps required to obtain information that could be used to distinguish each user, we investigated the classification rate using gait samples composed of steps in amounts ranging from one step (*k* = 1) to three steps (*k* = 3). Table 1 presents the total number of gait data samples according to the value of *k* with the number of training samples and test samples. When composing a data sample with an individual step (*k* = 1), the total number of data samples was 2295. When *k* = 2 and *k* = 3, the total number of data samples was 1144 and 759, respectively. To examine the identification performance as the value of *k* changed, we randomly selected 700 samples among all experiments for different *k* values, of which 42 training data samples were used to construct the NLDA feature space, and the remaining 658 samples were used for testing. To increase statistical confidence, we repeated the above procedure 25 times and calculated the average identification rate. From the samples, 700 samples were randomly selected; 42 samples (three per subject) were used for training; and the remaining 658 samples were used for testing. To ensure reliability, we repeated the above-mentioned process 25 times and used the average identification results. The one-nearest-neighborhood rule from the single- and multi-modal features was used as a classifier for user identification considering the Euclidean distance [36].

**Figure 4:**
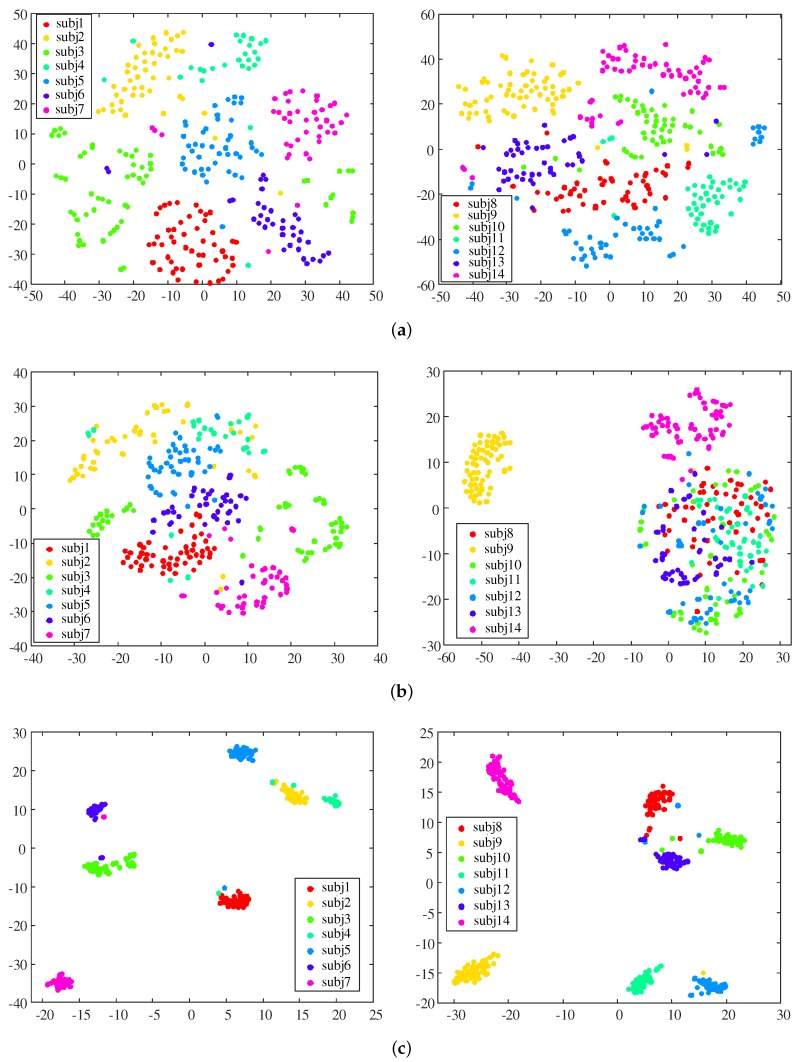
Distribution of individual step samples in each vector space: (**a**) input space of pressure data, (**b**) input space of acceleration data, and (**c**) multi-modal feature space.

Figure 4 shows the two-dimensional distribution of data samples from individual steps in the input data space ($x^{P}$ and $x^{A}$) and the multi-modal feature space ($y^{Mul}$) for the 14 subjects. To visualize the high-dimensional data in a plane, we used the t-distributed stochastic neighbor embedding [37], which performs nonlinear dimensionality reduction and is widely used in machine learning applications. In the sub-figures, each color represents an individual subject, and the points represent the data samples of individuals. Figure 4a,b shows that samples were clearly clustered by subject in the multi-modal feature space compared to the input data space. The clustering improvement by feature extraction was especially prominent in the acceleration data. In the multi-modal feature space, the variance of a subject cluster was much smaller than that in the input space of acceleration data.

**Figure 5:**
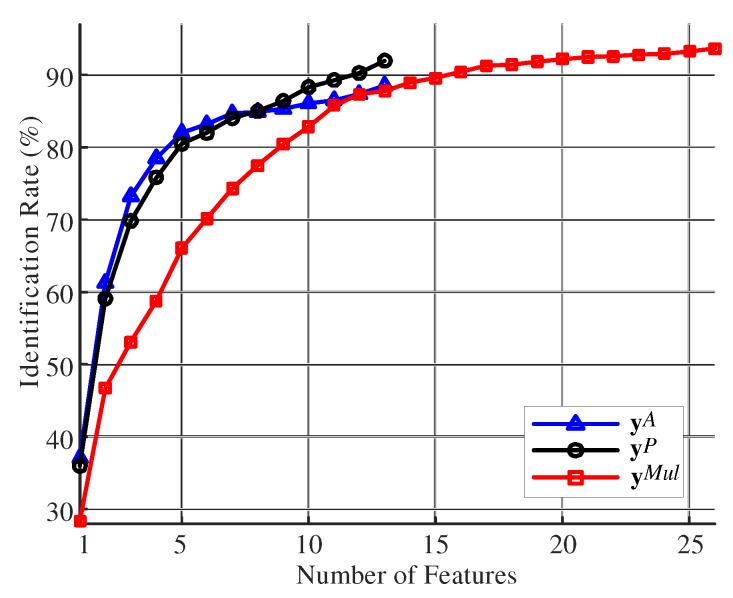
Identification rates for various dimensions of the feature space.

Figure 5 shows the user identification performance for each step. The multi-modal features ($y^{Mul}$) provided better identification performance than the single-modal features ($y^{P}$ and $y^{A}$ [38]). Moreover, the identification rate using $y^{Mul}$ increased gradually with the dimension of the feature space, but it saturated at around 20 dimensions. Hence, sequential feature selection from discriminability evaluation was effective at constructing a multi-modal feature vector for user identification. On the other hand, the single-modal features obtained from pressure data ($y^{P}$) retrieved better identification performance than that obtained from acceleration data ($y^{A}$). Hence, individual gait patterns distinguishing persons were better represented by the distribution of contact points of the soles during walking.

**Figure 6:**
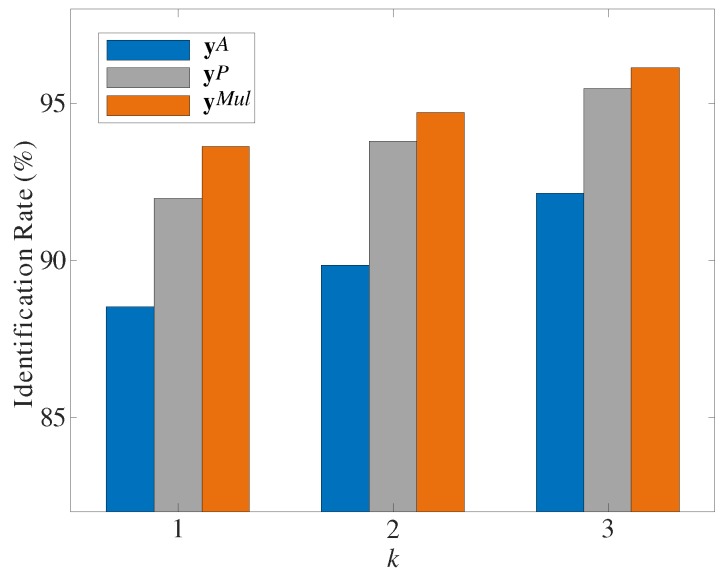
Identification rates for different *k*.

To determine the minimum number of steps necessary to extract gait information for accurate user identification, we evaluated the identification performance when the gait sample was constructed with one (k=1), two (k=2), and three (k=3) consecutive steps. When k=1, the number of samples was 2295, and when k=2 and k=3, the numbers of samples was 1144 and 759, respectively. Figure 6 shows the identification rate according to *k*, where the identification performance improved with *k*, as expected, for both single- and multi-modal features. Therefore, the more steps a single sample contained, the higher the discriminability of the feature. For every *k*, the multi-modal features provided better identification performance than the single-modal features, reaching above 93% identification accuracy at the lowest k=1. This may be given by the complementarity between the characteristics of gait data from different sensors, producing a synergetic effect that provides richer features for user identification, even from few available data samples.

## 5. Discussion

Since gait patterns have characteristics that are unique to each individual, gait pattern analysis can be used as a biometric to identify an individual. The contribution of our work is proposing a method for constructing a multi-modal feature space that is effective for user identification from gait data obtained from various wearable sensors. Most existing studies on the walking patterns of individuals for the purpose of user identification have taken gait videos with cameras and have analyzed them using a computer vision technology. However, video-based analysis methods have limitations on data acquisition, such as being limited to a specific space with an installed camera or requiring an uncrowded space to prevent occlusion. In addition, these methods for gait analysis require the cooperation of the user, as the user should walk in front of the camera for a while. Due to these constraints, video-based gait analysis methods have limited applicability in various fields outside of specific uses. Meanwhile, gait analysis methods using wearable devices, such as IMU sensors and smart insoles, have also been proposed. However, they have attempted a basic classification of several types of walking, and some methods still require the cooperation of the user, such as attaching the sensor to a specific part of the body.

The proposed method effectively extracted individual gait characteristics from the gait data measured using the wearable sensors and showed excellent user identification performance even with a small amount of computation. In particular, the proposed method used sensors mounted on an insole used in everyday life; hence, it did not require special cooperation from users for data acquisition. In addition, the data could be easily measured at any time while wearing shoes, allowing analysis of accumulated data over time. This can improve the reliability of security when applied to security systems, such as door control, because it can prevent being deceived by an impersonated gait pattern where the user’s walking style changes instantaneously.

Many methods for classifying data have also been developed, including deep learning-based approaches that have received much attention recently. However, although deep learning methods have shown excellent classification performance in various fields, massive datasets should be obtained for training. In addition, although lightweight deep learning methods [39,40,41] are being studied, their computational burden is still too high to be used in mobile/wearable devices. Therefore, in this paper, we used the NLDA method, one of the discriminant analysis techniques, which has shown good performance in the classification of high-dimensional data. The NLDA method is especially effective when the data dimension is large compared to the number of data samples where sufficient null space of the covariance matrix is secured. As insoles for both feet generated 16 pressure and six acceleration signals in real time, the obtained walking data were high-dimensional, and thus, we extracted discriminant features for user identification by using NLDA. The proposed gait classification using NLDA can even work on mobile devices without a graphics processing unit. The flexibility of the proposed method for applicable use environments and available devices is a significant advantage not only for the use of biometrics, but also for a wide range of applications, such as behavioral analysis through long-term observation and the diagnosis of neurologic disorders and musculoskeletal diseases.

## 6. Conclusions

We proposed a method for user identification based on discriminant analysis from gait data measured by multi-modal sensing on a smart insole. As the proposed method used a wearable device, it can be applied with less environmental constraints and lower computational burden than methods relying on video processing. In addition, as acquiring data through insoles was not limited by the activities of the users, our method had high scalability. The proposed method consisted of data preprocessing, discriminant analysis for single-modal data, construction of multi-modal feature vector, and user identification. Single-modal features were extracted using NLDA.The multi-modal feature vector was constructed by evaluating the discriminative power of each feature based on its Laplacian score. We used a commercial smart insole, FootLogger, for data acquisition. The user identification results on walking data acquired by pressure sensors and accelerometers from 14 adults confirmed that identification using multi-modal features integrating the sensing modalities outperformed identification using single-modal features. Although deep learning methods have shown excellent classification performance in various fields, massive datasets should be obtained for training. In future developments, we will measure walking data from more people and study more advanced user identification techniques based on multi-modal deep neural networks. We will also evaluate user identification for various gait types such as running and climbing, besides further investigating walking. Furthermore, we will aim to improve the user identification performance by considering data measured in various environments during execution of activities of daily living and combining our analysis and methods with gait type classification.

## Figures and Tables

**Table 1:** The total number of gait data samples according to the value of *k* with the number of training and test samples.

*k*	Total Number of Gait Samples	Number of Training Sample	Number of Test Sample
1	2295	42	658
2	1144	42	658
3	759	42	658
